# Salinity effects on water potential and the normalized difference vegetation index in four species of a saline semi-arid ecosystem

**DOI:** 10.7717/peerj.12297

**Published:** 2021-10-25

**Authors:** Hebert Hernán Soto Gonzáles, Ofelda Peñuelas-Rubio, Leandris Argentel-Martínez, Aurelio Leyva Ponce, María Hermelinda Herrera Andrade, Mirza Hasanuzzaman, Jorge González Aguilera, Paulo Eduardo Teodoro

**Affiliations:** 1 Escuela Profesional Ingeniería Ambiental, Universidad Nacional de Moquegua, Ciudad Pachoca, Ilo, Perú; 2 Departamento de Ingenierías, Tecnológico Nacional de México/Instituto Tecnológico del Valle del Yaqui, Bácum, México; 3 Department of Agronomy, Sher-e-Bangla Agricultural University (SAU), Dhaka, Bangladesh; 4 Department of Crop Scienc, Federal University of Mato Grosso do Sul, Chapadão do Sul, Mato Grosso do Sul, Brazil

**Keywords:** Electric conductivity, Stress intensity, Bursera, Parkinsonia, Prosopis, Atriplex, Halophytes, Xerophytes

## Abstract

This study was carried out during January 2020–December 2020 in a semi-desert ecosystem in southern Sonora, Mexico, to determine the annual and daily variations in water potential and the normalized difference vegetation index (NDVI) of *Bursera fagaroides* Engl., Monogr. Phan., *Parkinsonia aculeata* L., Sp. Pl.; *Prosopis laevigata* (Humb. & Bonpl. ex Willd.), and *Atriplex canescens* (Pursh) Nutt. Soil electrical conductivity, cation content, and physical characteristics were determined at two depths, and water potential (ψ) was measured in roots, stems, and leaves. The daily leaf ψ was measured every 15 days each month to determine the duration of stress (hours) and the stress intensity (SI). The electrical conductivity determinations classified the soil in the experimental area as strongly saline. A significant difference was noted in electrical conductivity between soil depths. The four studied species showed significant gradients of ψ in their organs. In this soil, all four species remained in a stressed condition for approximately 11 h per day. The mean SI was 27%, and *B. fagaroides* Engl., Monogr. Phan. showed the lowest value. The four species showed increased NDVI values during the rainy months, with *P. laevigata* (Humb. & Bonpl. ex Willd.) and *Parkinsonia aculeata* L., Sp. Pl. showing the highest values. The capacity for ψ decrease under saline conditions identified *A. canescens* (Pursh) Nutt., *B. fagaroides* Engl., Monogr. Phan. and *P. aculeata* L., Sp. Pl. as practical and feasible alternatives for establishment in saline soils in southern Sonora for purposes of soil recovery and reforestation.

## Introduction

Among the adverse conditions occurring in natural and agricultural ecosystems worldwide, soil salinity is a factor that strongly limits plant productivity (Abbas et al., 2013; Zhang et al., 2020; Helaly et al., 2016). Salinization is an accumulation of water-soluble salts in the soil, and it therefore entails more than just NaCl accumulation. Several other cations and anions can accumulate to high levels in the soil (Shao et al., 2019), including the cations potassium (K^+^), magnesium (Mg^2+^), and calcium (Ca^2+^) and the anions chloride (Cl^−^), sulfate (SO_4_^2−^), carbonate (CO_3_^2−^), and bicarbonate (HCO_3_^−^) (Alvarez-Acosta et al., 2018). Regions with high salt concentrations have water potentials (ψ) that become very low. Therefore, plants cannot take up water, even if water is present in the topsoil (Adams et al., 2017).

Salinity in a soil can affect plant growth because a reduction in the soil osmotic potential decreases water and nutrient availability. Salinity can also induce ion-specific effects, nutritional imbalances, and ion toxicity, thereby affecting normal plant metabolism, vegetal vigor (Shahbaz & Ashraf, 2013), and the normalized difference vegetation index (NDVI) (Bian et al., 2021). Some species respond to soil salinity conditions by activating mechanisms such as ion inclusion and accumulation in organs (Helaly et al., 2016; Ordoñez et al., 2018) and/or accumulation of osmotically active compounds to reduce the osmotic potential (and consequently ψ), thereby guaranteeing water uptake and plant survival (De Sedas et al., 2019). For this reason, the study of salinity responses has great biological and ecological significance.

In northwestern Mexico, and particularly in the coastal areas of the Sea of Cortés, several adverse abiotic factors, including drought (Devora-Isiordia, Valdez-Torres & Granillo-Moreno, 2018), thermal variations (Argentel-Martínez et al., 2019a), and salinity occur concomitantly and limit plant performance and survival. The expected scenarios of climate change for this region include a significant temperature increase (Copping et al., 2018), which would further affect the physiological performance of the existing plant species. Many of these species have not been able to adapt to changes in climatic and edaphic conditions; therefore, a detailed study of the existing species, even under adverse conditions, can serve as a reference indicator for future reforestation programs (Ash, Givnish & Waller, 2017). Species in ecosystems affected by abiotic stresses can often show little diversity, but the scientific and producer communities sometimes ignore that the species distribution is showing evidence of natural adaptation.

The study of these species can identify alternative species that can increase diversity in ecosystems and contribute to soil improvement and conservation. Among the most frequent species occurring in the semi-desert region of southern Sonora in fragile and degraded ecosystems are *Bursera fagaroides* Engl., Monogr. Phan., *Parkinsonia aculeata* L., Sp. Pl.; *Prosopis laevigata* (Humb. & Bonpl. ex Willd.), and *Atriplex canescens* (Pursh) Nutt. These species are characterized by morphological parameters that support adaptation to the edaphic and climatic conditions of the region (Bradley & Colodner, 2019). Therefore, these species can be important experimental models for ecophysiological and forestry studies.

The monitoring of physiological variables, such as ψ, can be used to understand the stress intensity experienced by plants and its effects on the foliar organs. The first steps in this type of analysis are therefore to determine the physicochemical characteristics of the soil at different depths, to establish the way that plants achieve water and nutrient uptake, to follow leaf area development, and to determine the vegetation index (*e.g*., the NDVI). In plants, the root system functionality is reflected in leaf area performance (Chen et al., 2019). High vegetal vigor (high NDVI) indicates that the roots have explored sufficient depth to obtain water and nutrients and support the transpiratory demand (Blum, 2017).

This relationship between root functionality and leaf area development is particularly important in semi-desert regions where growth conditions are adverse and plants must make metabolic and even molecular adjustments for survival (Hof, Dymond & Mladenoff, 2017). The aim of the present work was to evaluate ψ and the variability in NDVI variability in four species common to the saline semi-arid ecosystem in southern Sonora, Mexico. The main scientific question of the present study was: Do plants like *Bursera fagaroides* Engl., Monogr. Phan., *Parkinsonia aculeata* L., Sp. Pl.; *Prosopis laevigata* (Humb. & Bonpl. ex Willd.), and *Atriplex canescens* (Pursh) Nutt. show a significant correlation between their ability of to reduce their ψ under high salinity conditions and their NDVI?

## Materials & methods

**Figure 1:**
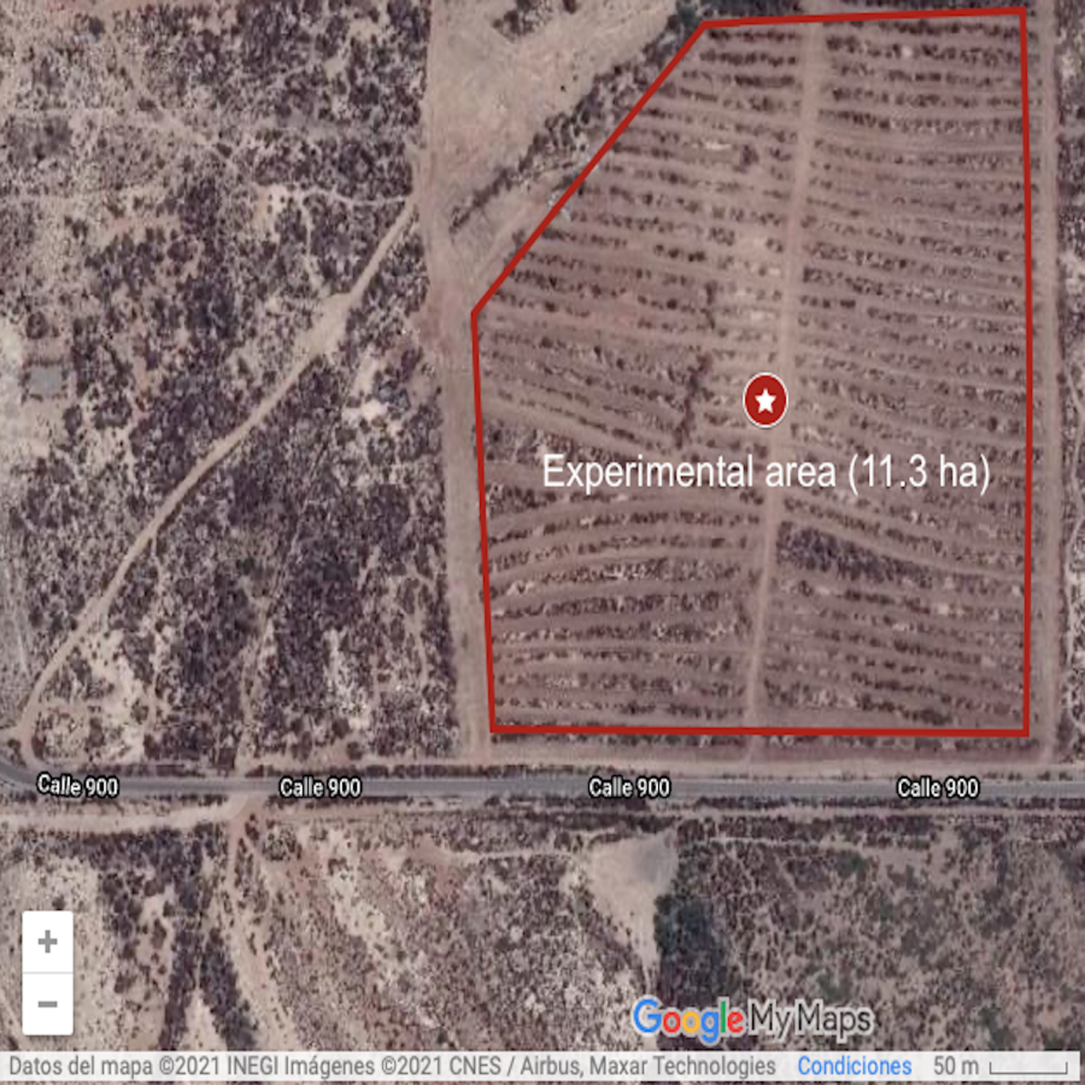
Satellite image, taken from Google Earth, 2020 corresponding to Block 937, Bahía de Lobos, Sonora, Mexico. ©2021 INEGI, Google Maps.

The experiment was carried out in Block 937 (27°35.6′27″N, 110°39′87″W) of the Yaqui Valley, Sonora, Mexico during January–December 2020 (Fig. 1). The sampled area was 11.3 ha, and 24 randomized points were taken at two depths in the soil (0–30 and 30–60 cm) for physico-chemical characterization. According to IUSS Working Group WRB (2015) this soil classifies as Gypsisol.

### Climate variables in the experimental site

**Figure 2:**
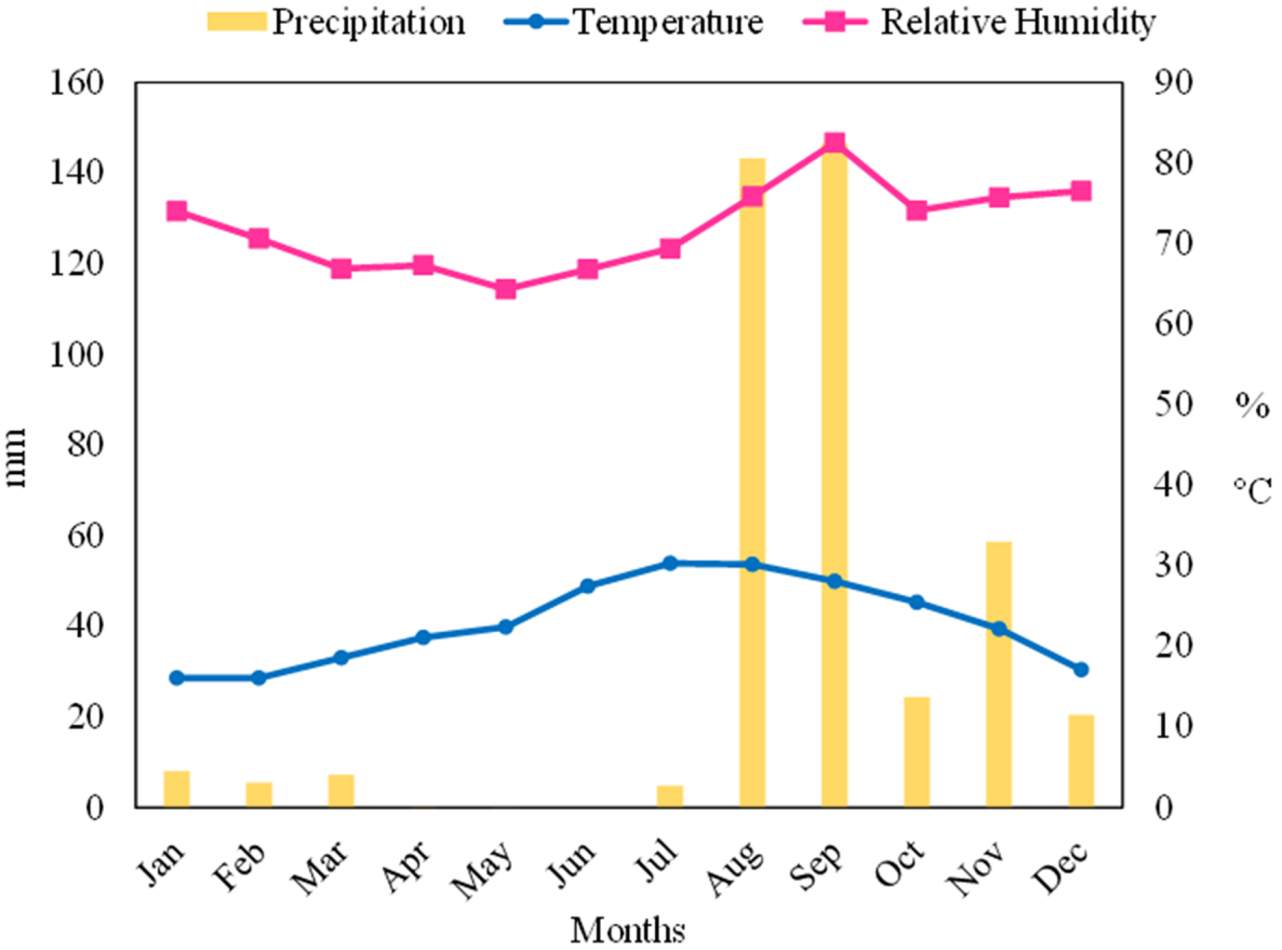
Main climatic variables in block 937 of Bahía de Lobos, Sonora, Mexico during 2020. Hydrothermal coefficient: HTC = 0.2. Historical data are available on the page: http://www.siafeson.com/remas2/index.php/tablero.

For the monitoring of the climatic variables, data registered in the agroclimatic Experimental Station were taken. During 2020, the relative humidity was between 65% and 84%, due to the proximity to the Sea of Cortéz coast (Fig. 2). The precipitations were only significant during August–December, being the highest accumulated in August and September with approximately 140 mm. The temperature in this region ranged between 18–30 °C. The hydrothermal coefficient value calculated during 2020 in the experimental area was HTC = 0.2, classifying it as very dry region (Evarte-Bundere & Evarts-Bunders, 2012).

### Species of major dominance at the experimental area

**Figure 3:**
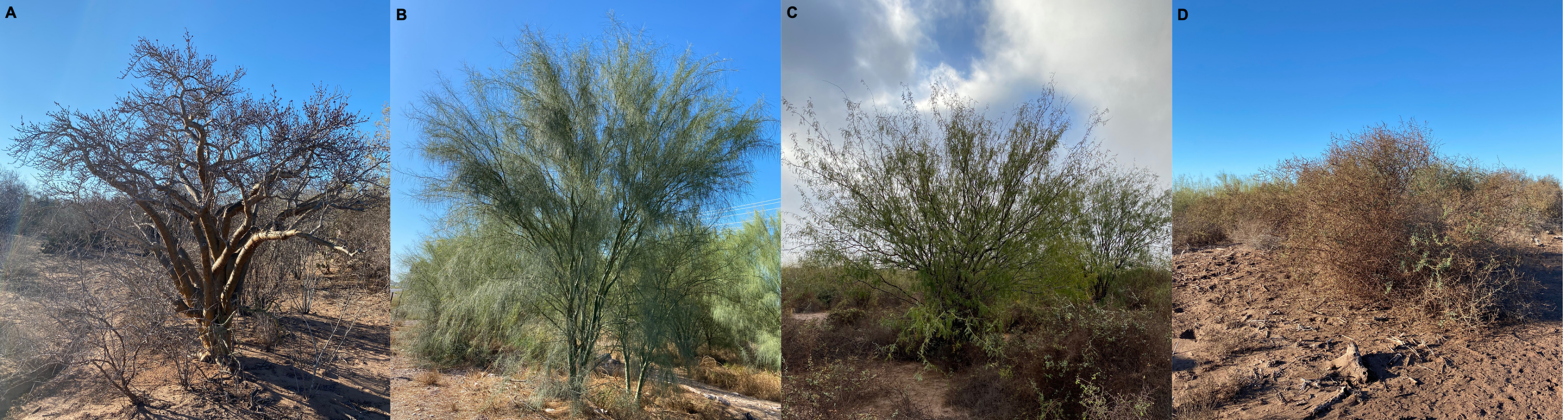
Imagen of *B. fagaroides* Engl., Monogr. Phan. (A), *P. aculeata* L., Sp. Pl (B), *P. laevigata* (Humb. & Bonpl. ex Willd.) (C) and *A. canescens* (Pursh) Nutt. (D). Photo credits: Ofelda Peñuelas-Rubio, 2020.

*Bursera fagaroides* Engl., Monogr. Phan., *Parkinsonia aculeata* L., Sp. Pl.; *Prosopis laevigata* (Humb. & Bonpl. ex Willd.), and *Atriplex canescens* (Pursh) Nutt. were selected. *Bursera fagaroides* Engl., Monogr. Phan (Fig. 3A) belongs to the Burseraceae family, comprising trees and shrubs. It is classified as a drought tolerant species (Rzedowski, Medina & de Rzedowski, 2005).

*Parkinsonia aculeata* L., Sp. Pl. (Fig. 3B) and *Prosopis laevigata* (Humb. & Bonpl. ex Willd.) (Fig. 3C) belong to Fabaceae family, which is representative of the xerophilous scrub of northwestern Mexico (Standley, 1922). *P. aculeata* L., Sp. Pl. is an evergreen, thorny, thin-bark tree species that is distributed in arid and semi-arid areas of Mexico. This specie can tolerate prolonged periods of drought since its stem is photosynthesizing (Giraldo, Cox & Hasenhüttl, 2015). *Prosopis laevigata* (Humb. & Bonpl. ex Willd.) “mesquite” is a species with multiple uses, since all parts of the plant are susceptible to be used. It grows as a tree or shrub with compound leaves and paired spines. Under its canopy, soil fertility and humidity tend to have higher values than in open areas (Bernal-Ramirez et al., 2019).

*Atriplex canescens* (Pursh) Nutt. (Fig. 3D) is a shrub belonging to Amaranthaceae family, it has a highly variable shape and is a common plant of the succession of recently turned soils and active sand dunes. The presence of *A. canescens* (Pursh) Nutt. indicates bad soil conditions (Segura-Castruita et al., 2014).

### Observed variables

Water potential was measured in roots, stems and leaves at 10:00 h, every 14–15^th^ of January, March and May, 2019. This was the period of less precipitations in the region. For measurements, right after collection, the samples were placed in the Schollander pressure pump (PMS-100; PMS Instrument Company, Albany, OR, USA).

In addition, leaf water potential measurement was carried out at: 3:00, 6:00, 10:00, 12:00, 15:00, 18:00, 21:00 and 23:00 h, to evaluate the day time of greatest variation in the four species using the same pressure pump. With such data the amount of hours under stress (SH) was calculated.

Stress intensity (SI) was determined according to Fernandez (1993) taking as normal water potential (ψ normal) the average from 23:00 to 6:00 h, and the stressed water potential (ψ stress) average which was significantly lower than the normal water potential (Eq. (1)). From this reasoning, the number of hours that the plants remained stressed was also determined.

(1) $${\rm SI} = (\Psi_{\rm stress}/\Psi_{\rm normal})$$

### NDVI measurements in the evaluated species

During 2020, every month, the normalized difference in vegetation index (NDVI) was measured with a portable sensor (Green Seeker, Trimble™ brand, Sunnyvale, CA, USA) (Govaerts & Verhulst, 2010). For each species six averages measurements at 0.60 m high from plant canopy were taken, according to the sensor reference. The measurement of this variable gives an approximation to the nutritional conditions of the plants and the possible incidence stress. Greater values of the NDVI (−1<NDVI>1) represent a better nutritional status of plants (Inman, Khosla & Mayfied, 2005).

### Statistical analysis

After testing the compliance of the theoretical assumption of normality of water potential data (Kolmogorov, 1933) an analysis of variance of double classification, with factorial arrangement, based on a linear model of fixed effects was done (Fisher, 1937). Tukey’s honest significant test was applied for mean comparison (Tukey, 1960). The statistical indicators standard error of the mean (SE), coefficient of variation (CV) and coefficient of determination without adjustment (R^2^) were determined. For EC comparison between soil depths, a theoretical distribution of t-Student test was used for a significance level of 5% (Gosset, 1917). Finally, Pearson’s correlation coefficient (r) between ψ-NDVI, and SH-SI were determined for each of the studied species.

For all data analysis the professional statistical package STATISTICA (TIBCO Software Inc., Palo Alto, CA, USA), version 12.0 for WINDOWS was used.

## Results

### Soil physico-chemical characteristics

The electrical conductivity (EC) was higher at the 0–30 cm depth than at the 30–60 cm depth, and the differences between the EC values were highly significant (t value = 14.8913; *p* = 0.0001). Although the sample standard deviation was low for the 24 sampled points, the coefficient of variation was high (greater than 21%) at the two depths. The greatest variability was found at 0–30 cm. No significant correlation was determined between the EC values at the sampled depths (*p* = 0.05632) (Table 1). According to Ivushkin et al. (2019), the soil in the study area is classified as strongly saline and represents a soil type expected to limit the normal development and productivity of glycophyte agricultural and forestry species.

**Table 1:** Electrical conductivity at depths of 0–30 and 30–60 cm. [N, Sample size; SD, standard deviation; SE, standard error; CV, coefficient of variation; r, correlation coefficient].

Depths (cm)	Valid N	Mean dS m^−1^	Minimum	Maximum	SD	SE	CV	r
0–30	24.00	8.09**	5.60	11.10	1.64	1.28	0.26	
30–60	24.00	3.05	1.60	5.30	1.12	1.06	0.22	0.58

**Note:**

** Significant difference by Student t-test at *p* < 0.001.

The sampled area has a high percentage of sand and silt (48% and 35%, respectively, on average). This condition favors the quickest desiccation, but the proximity of the aquifer to the surface favors the capillary rise of water and salts. The pH (pH 7.8, on average) is alkaline and the soil has a very low percentage of organic matter (OM) due to the scarce vegetation (OM < 0.2) This indicates a very poor soil in terms of organic natural fertility, which will decrease water maintenance as well as microbial diversity. The saturation percentage values confirmed the alkalinity, although CaCO_3_ percentage was low (Table 2).

**Table 2:** Main characteristics of the soil at depths of 0–30 cm and 30–60 cm. Acidity or basicity index (pH). Sand, silt, clay, organic matter (OM) and calcium carbonate (CaCO_3_) percentages.

Depths (cm)	pH (in H_2_O)	Physico-chemical analysis (%)					
		Sand	Silt	Clay	OM	Saturation	CaCO_3_
0–30	7.87	49.44**	36.69	13.87**	0.07	42**	2
30–60	7.89	47.27	35.62	17.11	0.04	37	1.9

**Note:**

** Significant difference by Student t-test at *p* < 0.001.

The soil also has a low N content (1.23 on average); however, the presence of legume species indicates the presence of a certain amount of N due to symbiosis with nitrifying bacteria (Alexander, Mishra & Jha, 2019). An increase in legumes in this soil would benefit soil conservation and N availability. The soil showed a null P content, low K content, and high Ca^2+^ and Na^+^ values (Table 3). Overall, the soil analysis indicated poor mineral composition, which represented a further limiting condition for plant growth.

**Table 3:** Main chemical characteristics of the soil at depths of 0–30 cm and 30–60 cm. [N, Nitrogen; P, Phosphorus; K, potassium; Mg, magnesium; Na, sodium].

Depths (cm)	Cations (kg ha^−1^)					
	N	P	K	Mg	Ca	Na
0–30	1.78**	00	3.32*	4.26**	2.18*	17.21**
30–60	0.79	0.0	2.17	2.85	1.82	15.85

**Note:**

Asterisks (* and **) represent significant difference Student t-test at *p* < 0.05 and *p* < 0.001, respectively.

### Water potential variability among species and vegetal organs

Highly significant differences were noted between the mean ψ of the four species and between organs; therefore, the null hypotheses for these two sources of variation are rejected. A significant interaction was also found between species and organ factors (Table 4). The calculated standard error was ±0.03, the standard deviation of treatments was s = 0.22 MPa, the general mean of treatments was x̄ = −4.2 MPa, and the coefficient of variation of treatments was CV = 5.2%.

**Table 4:** Analysis of variance of water potential of four species in Bahía de Lobos, Sonora, Mexico. (SS, Sum of squares; DF, degree of freedom; MS, Medium square; p, probability).

Sources of variation	SS	DF	MS	F	p
Intercept	644.3136	1	644.3136	11,714.79	0.000002
Species	96.8231	3	32.2744	586.81**	0.000173
Organs	4.8022	2	2.4011	43.66**	0.000142
Species * Organs	1.2311	6	0.2052	3.73**	0.009190
Error	1.3200	24	0.0550		
Total	104.1764				
R^2^ species	0.93				
R^2^ organs	0.046				
R^2^ interaction	0.012				

**Note:**

Asterisks (* and **) represent significant differences for *p* < 0.05 and *p* < 0.01. R^2^: coefficient of determination without adjustment, for isolated sources of variation and for factor interaction.

The species factor accounted for 93% of the total variability found in ψ, while the organ factor and the interaction contributed only 4.6% and 1.2%, respectively, to the total variability, leaving 2% of the total variability attributable to experimental error. The linear fixed effects model used for the statistical processing was adequate, since 98% of the total variability was explained.

### Post-hoc comparison among the species and organs

**Figure 4:**
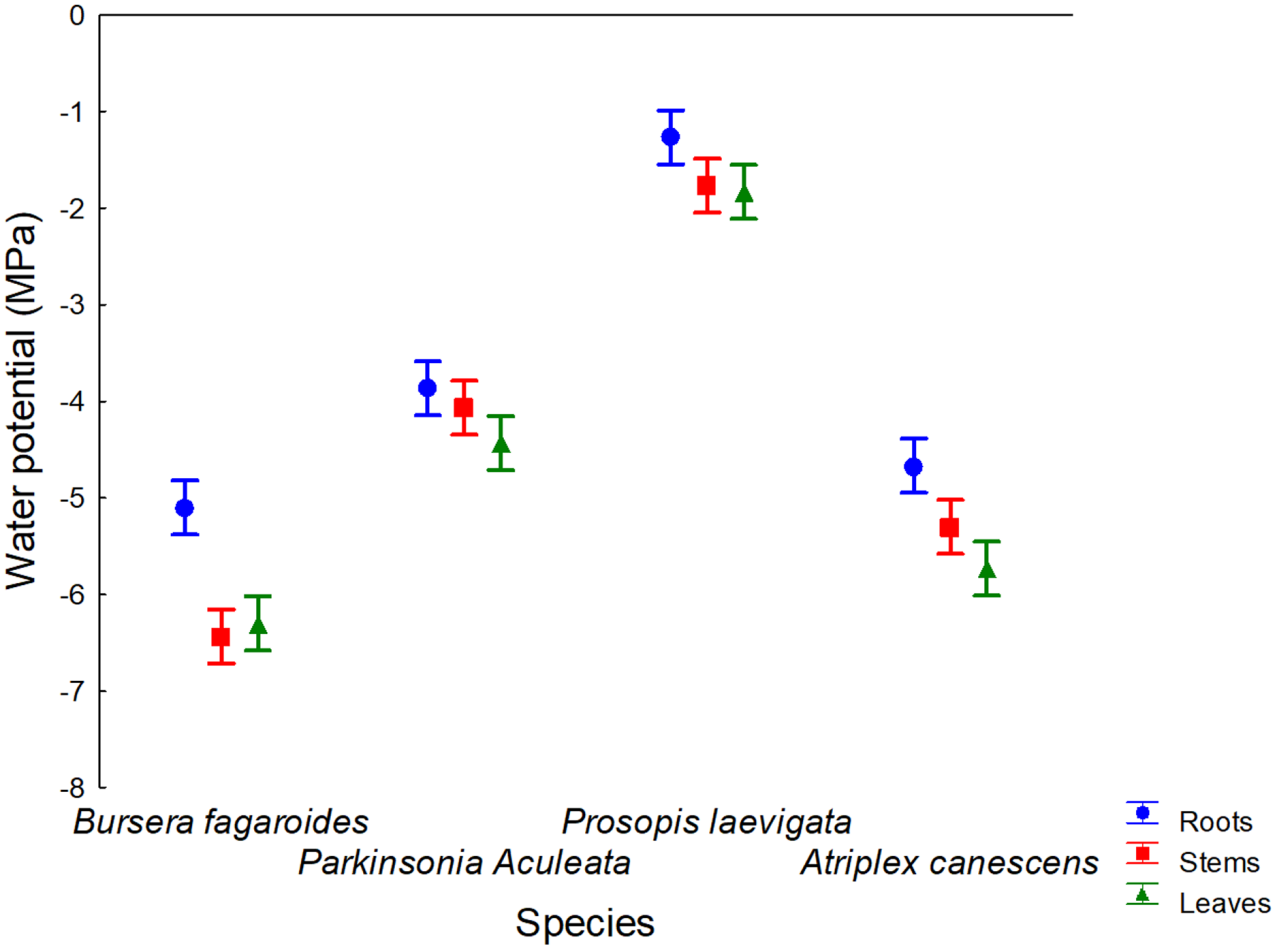
Root, stem and leaves water potential of four species in a saline soil of Bahía de Lobos, Sonora, México, during 2020. Rectangular bars represent standard deviation of means.

The four evaluated species showed a ψ gradient from the root to the leaves (Fig. 4). This finding confirmed the capacity of these species to guarantee water uptake in saline soils (Blum, 2017), where salts are an osmotic component that diminish soil water availability and limit the survival of other species.

The lowest average ψ values were found in *B. fagaroides* Engl., Monogr. Phan. (−5.9 MPa), *A. canescens* (Pursh) Nutt. (−5.2 MPa) and *P. aculeata* L., Sp. Pl. (−4.1MPa), indicating that these species were the most tolerant to salinity in the present study. The ψ value for *P. laevigata* (Humb. & Bonpl. ex Willd.) was only −1.6 MPa.

### Daily water potential variation per species

**Figure 5:**
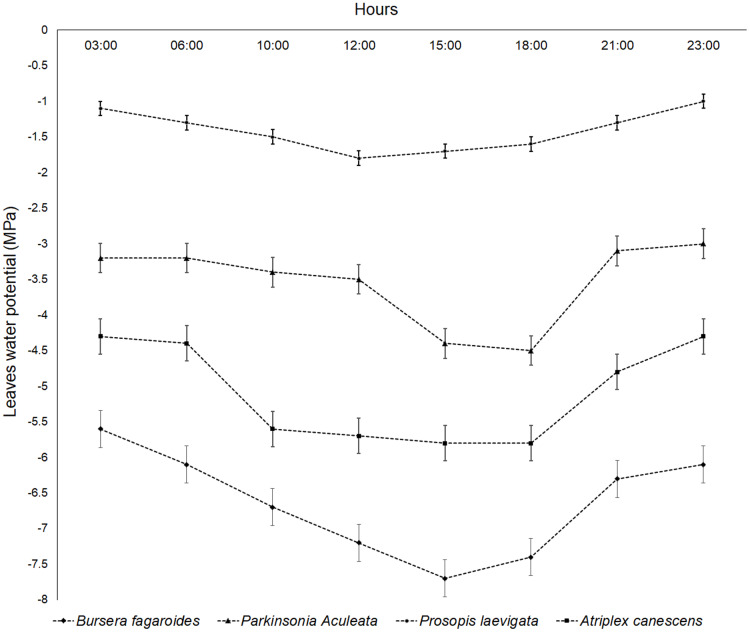
Daily leaves water potential (MPa) of four species in a saline soil of Bahía de Lobos, Sonora, México, during 2020. Rectangular bars represent standard deviation of means.

*P. laevigata* (Humb. & Bonpl. ex Willd.) had the minimum ψ value at 12:00 h, and its maximum variation in ψ was −0.7 MPa. This was the species that showed the smallest decrease in ψ due to the salinity condition. *P. aculeata* L., Sp. Pl. had a minimum ψ value between 15:00 and 18:00 h, and the ψ variation with respect to the measurement at dawn (6:00 h) was −1.3 MPa. *A. canescens* (Pursh) Nutt. significantly decreased its leaf ψ from 10:00 h and the ψ recovery occurred after 18:00 h. This species showed a ψ variation of −1.5 MPa with respect to the one measured at dawn (6:00 h) and was the species with the largest leaf ψ variation in the present study. *B. fagaroides* Engl., Monogr. Phan. had the greatest ψ variation with respect to the measurement at dawn (−1.6 MPa) (Fig. 5).

#### Average hours of stress: stress intensity

**Figure 6:**
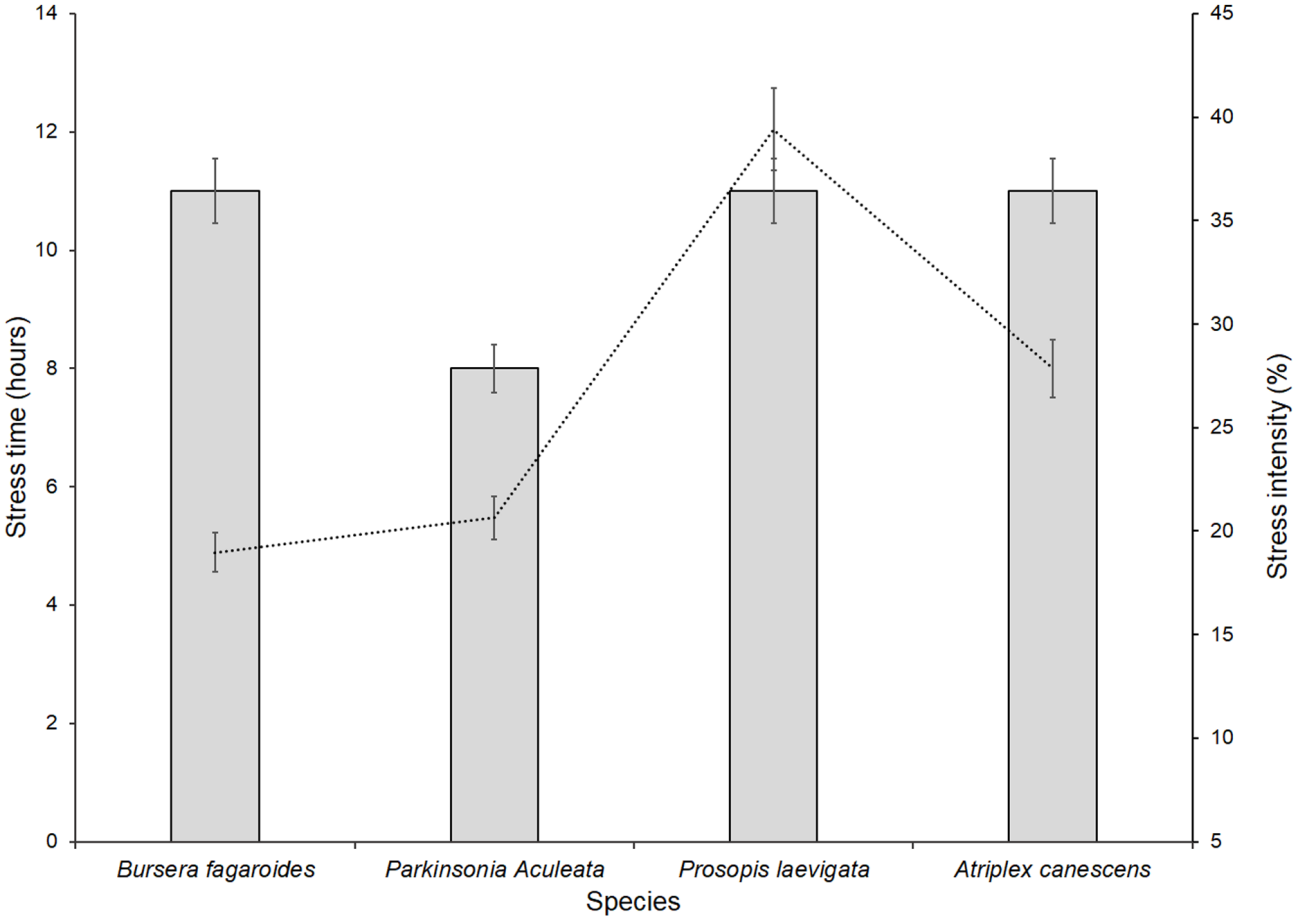
Stress intensity and amount of stressed hours of four species in a saline soil of Bahía de Lobos, Sonora, México, during 2020. Rectangular bars represent standard deviation of means.

*B. fagaroides* Engl., Monogr. Phan., *P. laevigata* (Humb. & Bonpl. ex Willd.), and *A. canescens* (Pursh) Nutt. were under stress conditions for 11 h per day, while *P. aculeata* L., Sp. Pl. was stressed for only for 8 h (Fig. 6). The calculated stress intensity among the four evaluated species averaged 27%. The species with the highest stress intensity were *P. laevigata* (Humb. & Bonpl. ex Willd.) and *A. canescens* (Pursh) Nutt. (greater than 25%), while *B. fagaroides* Engl., Monogr. Phan. and *P. aculeata* L., Sp. Pl. had average intensities of about 20%.

No significant correlation was noted between the time of stress and the stress intensity in *B. fagaroides* Engl., Monogr. Phan. (r = 0.44, *p* = 0.564642), *P. aculeata* L., Sp. Pl. (r = 0.49, *p* = 0.532828) and *A. canescens* (Pursh) Nutt. (r = 0.61, *p* = 0.503417), whereas *P. laevigata* (Humb. & Bonpl. ex Willd.) (Humb. & Bonpl. ex Willd.) did show a correlation (r = 0.97, *p* = 0.000174) between the 11 h stress condition and a stress intensity of 35%.

A positive and significant mean general correlation was found between ψ and NDVI (r = 0.71 *p* = 0.34671), showing that a greater decrease in ψ increased the water availability to maintain the plant leaf area alive and metabolically active. *B. fagaroides* Engl., Monogr. Phan. (0.844, *p* = 0.000462), *P. aculeata* L., Sp. Pl. (r = 0.89, *p* = 0.002711), and *A. canescens* (Pursh) Nutt. (r = 0.71, *p* = 0.004216) showed the highest correlation values.

### NDVI in the evaluated species

**Figure 7:**
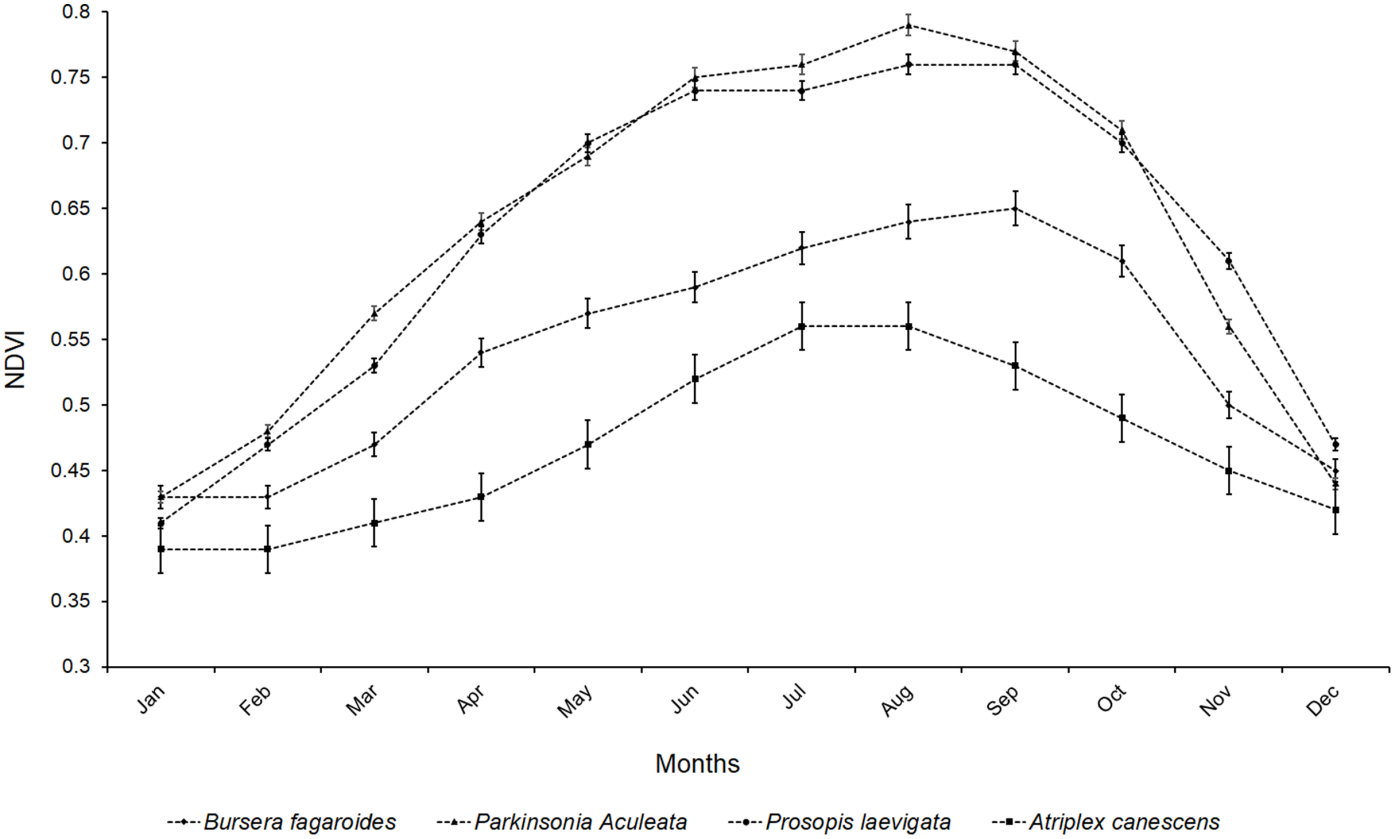
Mean values of NDVI of four species in a saline soil of Bahía de Lobos, Sonora, México, during 2020. Rectangular bars represent standard deviation of means.

The highest NDVI values were found in *P. aculeata* L., Sp. Pl. and *P. laevigata* (Humb. & Bonpl. ex Willd.), with no significant differences between the values throughout the year. This response indicated salinity tolerance and was supported by the maintenance of green foliage even at high salt concentrations. Lower NDVI values were found for *B. fagaroides* Engl., Monogr. Phan. and *A. canescens* (Pursh) Nutt., with highly significant differences between them. The salinity condition prevented the NDVI from exceeding a value of 0.8 in all four evaluated species. The highest values, found in *P. aculeata* L., Sp. Pl. and *P. laevigata* (Humb. & Bonpl. ex Willd.) (0.79 and 0.76, respectively), were obtained in the months of August and September. In *B. fagaroides* Engl., Monogr. Phan., the highest value occurred in September (0.65), whereas the highest value for *A. canescens* (Pursh) Nutt. was only 0.54 and was observed in July (Fig. 7).

## Discussion

High salinity in soils is a limiting factor for plant development due to the difficulty—and in extreme cases, the impossibility—it imposes in some plant species for water uptake. Salinity causes both ionic toxicity and osmotic stress in plants, and this situation reduces the vegetal cover and favors an increase in evaporation, due to the increased evaporative surface of soil exposure to direct radiation. This, in turn, causes soil salt concentrations to increase (Donohue et al., 2010), and is the most aggravating situation in semi-desert soils found in proximity to the coastlines (Woods, Swall & Zinnert, 2020).

Soils with EC greater than six dS m^−1^ in the saturation extract are not suitable for plant development. Therefore, salinity tolerance monitoring of the germplasm available in ecosystems with these salinity levels is an important process (Duberstein et al., 2020). Identifying intra- or interspecific variability is the first step in reforestation programs in saline soils (Woods, Swall & Zinnert, 2020).

The presence of organic matter in soil is a reflection of carbon capture and balance in soil as well as evidence of the leaf area carboxylation efficiency. Organic matter helps to maintain soil humidity and to stabilize soil surface temperatures (Carter, 2020); therefore, both organic and chemical fertility are important for plant growth and ecosystem functionality. For example, N is immensely important, as a lack of N will stunt leaf growth and hamper leaf functionality. In a general sense, the nutrients required by plants in the largest amounts are N, phosphorus (P), and potassium (K). Soils that are low in NPK, as at the study site, therefore support only low plant diversity and NDVI (Rahman, Ahmed & Ahmed, 2020). Saline soils are also generally low in organic matter (Sameen, Syed & Alvina, 2016); therefore, one way to mitigate salinity problems is by incorporating organic matter. Organic matter increases the negative charges in the soil, which promotes an increase in cationic exchange capacity (CEC) and is linked to improvements in the chemical properties of the soil (Srivastava, 2020).

Some studies carried out in Sonora show that salinity has been increasing even in agricultural areas. Comparison of the maps of salinity obtained in 1996 and 2001 shows that the surface mapped as saline in the Sonora region in 2001 was 122,754 ha, while in 1996 it was 120,778 ha (Pulido & González, 2010). The main causes for increased salinity have included the high doses of fertilizer applied to the established crops, the felling of the existing species in the periphery of crop fields that could have taken up the residual fertilizers applied to the crops, and the elimination of harvest wastes that otherwise could serve as organic matter and prevent evaporative water losses and salt concentration in the soil (Ojeda-Barrios et al., 2021). The maintenance and reforestation of halophytes and other plant species that show moderate tolerance to salinity stress conditions could be one key to slowing the rate of soil salinization in the Sonora region.

In nature, some plant species possess several adaptive features that facilitate their survival under saline conditions, and some of these plants are used for the remediation of salt-affected soils (Hasanuzzaman et al., 2014; Hasanuzzaman, Fujita & Shabala, 2018). Therefore, understanding the physiological mechanisms of plant adaptation to salinity is very important when considering the use of these types of plants for biosaline agriculture. Devi et al. (2016) developed an important assay using species of *Atriplex* and *Parkinsonia* for a process of phytoremediation of saline soil. Both genera were cultivated in soils having EC of eight dS m^−1^, and a significant reduction of EC in the soil was observed 6 months later due to ion extraction by plants. As a result, these types of plant species can be the key to salt phytoremediation and an appropriate approach to reforestation. Notably, both *B. fagaroides* Engl., Monogr. Phan. and *P. aculeata* L., Sp. Pl. have been reported as tolerant and moderately tolerant to saline stress in regions of Florida, according to Nellis (2015).

Exposure to salinity causes a loss of water from plant cells and a decrease in the cellular ψ. This decrease in ψ under salinity conditions has been reported as an important indicator of adaptation to saline stress in multiple crop and forest species (Langston, Kaplan & Putz, 2017). This variable can serve as an approximation of the osmotic adjustment capacity in different plant organs (Blum, 2017). Osmotic adjustment in many species occurs by the active absorption of cations and anions (Zhao et al., 2020) and their further compartmentalization, mainly in the leaves (Shekoofa et al., 2013). Some species avoid salt toxicity by synthesizing osmotically active compounds (Turner, 2017), such as proline, glycine betaine, glutathione, and others, to increase the osmotic pressure and decrease the osmotic potential and, consequently, ψ (Argentel-Martínez et al., 2019a).

Normally, plants show decreases in their foliar ψ at noon due to high transpiratory activity (Gebauer, Horna & Leuschner, 2012). This process is called the temporary wilting point. The recovery time for ψ in normal conditions of humidity in the soil is approximately 2 h (Loka et al., 2020). When the plant ψ decreases for longer than 4 h, this is considered a stress condition.

The studies carried out by Tardieu & Simonneau (1998) created a distinction between isohydric and anisohydric species. Isohydric species maintain a fairly constant minimum leaf ψ, as occurred in *P. laevigata* (Humb. & Bonpl. ex Willd.). By contrast, anisohydric species experience marked drops in leaf ψ, which have been hypothesized to promote earlier stomatal closure during water deficits. Anisohydric taxa are thought to be more vulnerable to hydraulic failure, as their stomata close later and plants reach more negative ψ values (Martinez-Vilalta & Garcia-Forner, 2017), as was observed in *P. laevigata* (Humb. & Bonpl. ex Willd.).

The responses of the four species could be related to the precipitation that occurred in the months of July, August, and September, as this would have decreased the salt concentration in the absorbent complex of the soil, facilitated better absorption of water and nutrients, and therefore promoted better foliar development. The NDVI is an important indicator of the nutritional state of plants (Stefen et al., 2016) and offers an approximation of the efficiency of use of N available in the soil (Allbed, Kumar & Aldakheel, 2014). The hypothesis could perhaps be corroborated with the stable isotope techniques applied in forest sciences (Argentel-Martínez et al., 2019b).

Salinity causes reductions in leaf area in several agronomic and forest species (Sun et al., 2018), and this may explain the low NDVI values observed in the present study. Leaf area reduction is a morphological modification by plants to avoid water loss by transpiration, but this adaptation also significantly reduces the photosynthetic activity (Swoczyna & Latocha, 2020). For this reason, both growth and productivity are commonly affected in saline ecosystems. Multiple species, and particularly woody xerophilic plants, have developed mechanisms to prevent water loss through transpiration (Eckert et al., 2019).

Plants with a high capacity for ψ decrease have a greater possibility of obtaining the water that is strongly retained in the soil capillaries. This condition allows higher NDVI; however, it also requires that the plant be able to evade Na^+^ cations, as these generally create nutritional interferences with N (Bulbovas et al., 2020) and generate toxicity that can lead to senescence and abscission and thereby reduce NDVI. The NDVI is therefore a precise indicator of good physiological and nutritional status in plants under salinity conditions (Bian et al., 2021).

Maintaining ψ is an important stress tolerance trait of plants, and plant survival in semi-arid and arid regions greatly depends on this ability (Hanin et al., 2016). The present study provides insight into salinity responses of the species that are naturally present in the semiarid region of southern Sonora. The fact that these plants can take up water under highly saline conditions reveals the activation of physiological and biochemical mechanisms that can be the key for reforesting programs. The proliferation of salt-tolerant tree species in fragile and degraded ecosystems should aid in increasing the biodiversity of microorganisms and plant species needed to regulate the carbon balance in the soil, thereby enhancing the amount of organic matter and opening up possibilities for growth and development of new species (Monteverdi, Lauteri & Valentini, 2008).

Conversely, soil conservation programs are still necessary in southern Sonora, since this region is located in the vicinity of the coastal zone. This is the main factor that contributes to the salinization and natural sodification as geological phenomena that increase the salt concentration in Sonoran groundwater (Swoczyna & Latocha, 2020).

## Conclusions

The four plant species studied here showed a ψ gradients in their organs that maintain the water column and allow survival in saline soils. In the Sonoran soil, the four species remained in a stressed condition for approximately 12 h (from 10:00–18:00 h) until recovery of ψ (from 23:00–6:00 h). According to their capacities to decrease their ψ under saline conditions and remain alive, *A. canescens* (Pursh) Nutt., *B. fagaroides* Engl., Monogr. Phan., and *P. aculeata* L., Sp. Pl. can be considered as practical alternatives for establishment in saline areas. All four studied species increased their NDVI values in the rainy months, but *P. laevigata* (Humb. & Bonpl. ex Willd.) and *P. aculeata* L., Sp. Pl. showed the highest values. Although *A. canescens* (Pursh) Nutt. and *B. fagaroides* Engl., Monogr. Phan. showed lower NDVI values, some other evaluated indicators confirmed their adaptation to salinity in this region, suggesting that these two species may also be valuable for reforestation programs in saline ecosystems.

## Supplemental Information

10.7717/peerj.12297/supp-1 Supplemental Information 1 Data of the evaluated variables in the saline ecosystem of Bahía de Lobos.

## Additional Information and Declarations

### Data Availability

The following information was supplied regarding data availability: The raw measurements are available in the Supplemental Files.
